# Advances in HIV Treatment and Vaccine Development: Emerging Therapies and Breakthrough Strategies for Long-Term Control

**DOI:** 10.1155/arat/6829446

**Published:** 2025-07-04

**Authors:** Olalekan John Okesanya, Racheal Ahuoyiza Ayeni, Promise Amadin, Ifeanyi Ngwoke, Blessing Olawunmi Amisu, Bonaventure Michael Ukoaka, Mohamed Mustaf Ahmed, Tolutope Adebimpe Oso, Shuaibu Saidu Musa, Don Eliseo Lucero-Prisno

**Affiliations:** ^1^Department of Public Health and Maritime Transport, University of Thessaly, Volos, Greece; ^2^Department of Medical Laboratory Science, Neuropsychiatric Hospital, Aro, Abeokuta, Ogun State, Nigeria; ^3^Department of Medical Laboratory Science, Chrisland University, Abeokuta, Nigeria; ^4^Department of Medical Microbiology, Department of Public Health Science, University College Hospital Ibadan, Ibadan, Nigeria; ^5^Department of Medical Laboratory Science, Edo State University Uzairue, Benin City, Nigeria; ^6^Department of Medical Microbiology and Parasitology, Bayero University Kano, Kano, Nigeria; ^7^Department of Medical Laboratory Science, State Hospital Ede, Ede, Osun, Nigeria; ^8^Community and Clinical Research Division, First On-Call Initiative, Port Harcourt, Nigeria; ^9^Faculty of Medicine and Health Sciences, SIMAD University, Mogadishu, Somalia; ^10^Faculty of Medicine, School of Global Health, Chulalongkorn University, Bangkok, Thailand; ^11^Department of Nursing Science, Ahmadu Bello University, Zaria, Nigeria; ^12^Department of Global Health and Development, London School of Hygiene and Tropical Medicine, London, UK; ^13^Research and Development Office, Biliran Province State University, Naval, Leyte, Philippines; ^14^Research and Innovation Office, Southern Leyte State University, Sogod, Southern Leyte, Philippines

**Keywords:** antiretroviral therapy, broadly neutralizing antibodies, gene editing, HIV, prevention strategies, vaccine development

## Abstract

Since its identification in 1981, HIV has posed a global public health challenge, witnessing transformative advancements in treatment and prevention. This review summarizes recent novel therapeutic and preventive approaches for long-term HIV control, management, and elimination, and how global collaboration and technological innovations may advance HIV control efforts. This study highlights the progress and challenges in HIV treatment, emphasizing the effectiveness of current antiretroviral therapy (ART) in suppressing viral replication, reducing transmission, and preventing end-organ damage. However, adherence remains a significant barrier due to pill burden, side effects, and psychosocial factors affecting patients. ART-related toxicities include neuropathy, hepatotoxicity, metabolic disorders, and neuropsychiatric effects. Long-acting ART (LA-ART) offers a promising alternative to daily dosing; however, challenges such as injection site reactions persist. Broadly neutralizing antibodies (bNAbs) have shown enhanced efficacy in viral suppression and immune response activation, offering potential for treatment and vaccine design. Innovative gene-editing tools, such as CRISPR–Cas systems, are being explored for their ability to excise or silence proviral DNA; however, their clinical application is limited by off-target effects and delivery challenges. Latency-targeting strategies like “shock and kill” and “block and lock” remain experimental with limited clinical success, and nanotechnology-based drug delivery systems offer targeted, sustained, and less toxic treatment options. Despite the challenges posed by the virus's rapid mutation rate and immune evasion mechanisms, novel vaccine approaches, such as mRNA technology, vector-based platforms, and epitope-targeting strategies, are being explored. In addition, artificial intelligence and machine learning are enhancing the design of vaccines, predictive modeling, and fast-tracking progress in this area. Socio-economic bottlenecks in HIV control, such as stigma, gender disparities, and inequitable healthcare access, exacerbate the epidemic, particularly in sub-Saharan Africa. Enhancing global collaboration, providing sustainable funding, and integrating emerging and innovative technologies are critical for advancing HIV prevention and management. Achieving an AIDS-free generation and ultimately eliminating the epidemic will depend on effectively addressing the social, structural, and scientific barriers that hinder progress in this regard.

**Trial Registration:** ClinicalTrials.gov identifier: NCT02120352, NCT02938520, NCT03639311, NCT03497676, NCT03635788

## 1. Introduction

HIV is a retrovirus that weakens the immune system by attacking the body's white blood cells or CD4+ cells. HIV is transmissible when bodily fluids such as breast milk, sperm, blood, and vaginal secretions come into contact with a site that permits HIV penetration [1]. HIV integrates into the genome of the host to become an HIV provirus upon invading target cells that express CD4 [2]. Since its discovery in 1981, HIV has become a major global public health issue [3]. In 2023, 39.9 million people were living with HIV (PLHIV) worldwide, with 20.8 million of them residing in Africa, including 1.4 million children aged 0–14 years. As of 2023, women and girls represented 53% of all PLHIV, with 86% aware of their HIV status. Since the onset of the epidemic, AIDS-related illnesses have claimed approximately 42.3 million lives, including 630,000 deaths in 2023 alone. In the same year, there were an estimated 1.3 million new HIV infections globally, with Africa accounting for 450,000 of these cases [4]. However, in some parts of Africa, 10%–20% of PLHIV are infected with two or more viral variants [5]. Combinations of antiretroviral therapies (ARTs) from multiple classes, such as reverse transcriptase inhibitors, protease inhibitors (PIs), integrase strand transfer inhibitors (INSTIs), capsid inhibitors, entry inhibitors, attachment inhibitors, and CD4-directed postattachment inhibitor monoclonal antibodies, are used to treat HIV infection. These therapies target different stages of the viral replication cycle [6]. While ART is effective at suppressing viral replication, it does not eliminate HIV or cure those already infected [7]. One promising immune-based approach for HIV treatment is the development of a vaccine. For an HIV vaccine to be effective, it must not only control viral replication and prevent rebound after ART is discontinued, but also trigger a stronger or qualitatively different immune response than that produced by natural infection [8].

Although combination antiretroviral therapy (cART) is a highly effective “reduce and control” technique, prolonged lowering of plasma viral loads to undetectable levels does not equate to viral eradication. Despite these significant advances, cART does not eliminate the virus from the body [9]. Furthermore, several severe non-AIDS events, including liver disease, cancer, cardiovascular diseases (CVDs), long-term peripheral and central nervous system (CNS) disorders, renal and metabolic abnormalities, and osteoporosis, are frequently linked to long-term cART treatment [10]. The rapid rate of mutation and recombination during viral replication is the biggest obstacle to the development of an effective HIV vaccine [11]. Therefore, regardless of clinical condition, infection duration, or CD4 cell count, all PLHIV should have an early HIV diagnosis and start ART [12]. HIV infection is now a clinically treatable chronic illness because of the development of powerful ARTs, which are now administered as a single pill once daily [9]. However, current ART regimens are not curative, and the lifelong burden of treatment, adherence issues, and long-term toxicities highlight the necessity for innovative therapies that can achieve durable viral suppression without daily medication [13].

Therapeutic strategies targeting long-term neutralization of the latent HIV reservoir have been investigated since the identification of latent HIV infection with limited transcription. These include chimeric antigen receptor T cells, gene-editing Clustered Regularly Interspaced Short Palindromic Repeats (CRISPR), vectored delivery of broadly neutralizing HIV antibodies (bNAbs), antibody-dependent cellular cytotoxicity (ADCC)-mediated bNAbs, shock and kill (latency reversion), and block and lock (transcriptional silencing) [14]. The global expansion of long-acting ART (LA-ART) toward more patient-friendly options and recent HIV vaccine trials highlights the limitations of immunological approaches [15]. Gaps in vaccine development necessitate the exploration of emerging therapeutic strategies for HIV treatment and prevention, while gene-editing technologies, such as CRISPR, represent a promising frontier for functional cures [15, 16]. Therefore, a comprehensive review is necessary to evaluate innovative strategies for long-term HIV viral control and eradication in humans. Thus, this narrative review aimed to summarize recent novel therapeutic and preventive approaches for long-term HIV control, management, and elimination and how global collaboration and technological innovations may advance HIV control efforts.

## 2. Methodology

We conducted a comprehensive literature search across PubMed, Scopus, and Google Scholar databases due to their broad and interdisciplinary coverage of peer-reviewed biomedical literature. These databases were deemed appropriate because of their high relevance in indexing both clinical and experimental HIV-related studies. Searches were conducted across these electronic databases using Medical Subject Headings (MeSH) keywords for PubMed and relevant keywords for other databases. The search on PubMed utilized MeSH terms such as “HIV,” “Human Immunodeficiency Virus,” “Vaccines,” “Therapy,” “Antiretroviral Therapy,” “ART,” “ARV,” and “Long-acting ARV,” which were combined in search strings using Boolean operators such as AND/OR to refine the search results. Studies were deemed eligible for inclusion if they discussed any evidence of emerging advances in HIV treatment, including recent LA-ART or vaccines undergoing therapeutic trials, with no limit on the publication year; however, priority was given to recent articles for the currency of data. We included a broad range of publication types, such as narrative reviews, including systematic and meta-analyses; perspectives; commentaries; editorials; opinion pieces; original research articles; intervention studies, including clinical and preclinical studies; and gray literature published in English, provided they contributed meaningfully to the discourse and were directly relevant to the study aim to ensure a comprehensive synthesis of current knowledge and insights on emerging HIV therapies. Studies that did not align with this theme but appeared in the search results were excluded from the analyses. This study was conducted as a targeted literature review rather than a systematic review to synthesize relevant and emerging evidence on innovative therapeutic strategies for HIV prevention and treatment. Two independent reviewers (OJO and NI) screened the articles, which were identified and selected iteratively based on their relevance to the review's objectives as they were encountered in the selected databases. Any discrepancies in the inclusion decisions were resolved through discussion and consensus with a third reviewer (MMA). Given the narrative nature and scope of this targeted review, a formal risk of bias assessment was not performed. This approach was deemed appropriate for capturing a wide range of evidence types, including peer-reviewed and gray literature. We employed a narrative synthesis using a thematic analysis approach to group the key ideas and findings into relevant themes. The extracted data were further discussed under appropriate headings to provide a clear and comprehensive overview of the study findings.

## 3. Overview of Current Approaches in HIV Treatment

### 3.1. ART

The National Guidelines for HIV Prevention, Treatment, and Care 2020 state that ART uses a combination of ARTs to treat HIV infections to enhance the quality of life, increase survival, reduce mortality and morbidity associated with the virus and related illnesses, and stop transmission [17]. ART diminishes inflammation caused by immunological activation linked to persistent HIV infection in addition to suppressing HIV. This suppression reduces the risk of end-organ illnesses, including neurological, cardiovascular, and renal conditions, which are prevalent among PLHIV [17, 18]. ARTs are divided into classes according to their mode of action, with each class focusing on a particular stage of the viral life cycle. Nucleoside/nucleotide reverse transcriptase inhibitors (NRTIs), such as tenofovir and emtricitabine, function as substrates for the elongation of reverse transcriptase chains by competing with host nucleotides. Non-nucleoside reverse transcriptase inhibitors (NNRTIs), such as efavirenz and rilpivirine (RPV), bind to a hydrophobic pocket close to the active site of HIV reverse transcriptase, inhibiting it and locking it in an inactive conformation [19]. Atazanavir and darunavir are PI therapies that stop the cleavage of Gag and Gag–Pol precursors by attaching to the active site of the HIV protease.

Although virions are created, they are not infectious and remain incomplete [20]. Entry inhibitors, such as maraviroc, block HIV entry into CD4+ cells by preventing entry mechanisms. There are three types of entry inhibitors: attachment inhibitors, which attach to glycoprotein 120 and prevent the virus from adhering to the cell; fusion inhibitors, which work against the viral protein GP41, preventing the virus from fusing with cellular membrane molecules; and chemokine receptor antagonists, which attach to fusion proteins such as CXCR4 and CCR5 [21, 22] HIV integrase inhibitors, also known as INSTIs, such as dolutegravir and bictegravir, prevent the virus from integrating by blocking the transfer of its DNA into the host cell's genome. Importantly, integrase inhibitors do not confer resistance to other ART regimens [23]. PIs bind to the active site of the HIV protease enzyme, blocking its activity and competing for proteolytic cleavage of Gag/Pol polyproteins in HIV-infected cells, producing immature, noninfectious virions [24] (Table 1).

### 3.2. Challenges and Limitations of ART

The chance of developing severe AIDS and AIDS-related diseases is considerably decreased by starting highly active antiretroviral therapy (HAART) early [28–31]. Some therapies are not currently accessible in conjunction with formulas, which results in a substantial pill load for patients who need to take three or four pills twice a day. However, many medications are administered orally once a day as co-formulated combination tablets. Patient adherence may be impacted by this, especially for patients who are struggling financially, have dysphagia, or live in environments with limited resources, which has been linked to several factors, including stress, depression, anxiety, ART duration, ART regimens and side effects, and sociodemographic and sociocultural characteristics [32, 33]. In addition to decreasing ART effectiveness, noncompliance raises death rates [34], lowers CD4 counts, and causes medication resistance [35].

Peripheral neuropathy and lactic acidosis can result from the mitochondrial damage caused by NRTIs. Additionally, some NRTIs can impair bone marrow function, leading to lipodystrophy and anemia [36]. Although tenofovir is usually well tolerated, it can lower bone mineral density and damage the kidneys. Patients with HLA-B 5701 mutation have been found to experience CD8-mediated hypersensitivity responses when using abacavir. Due to the hazards of hepatomegaly and pancreatitis, didanosine is rarely administered (Table 1) [37–41]. Rashes caused by NNRTIs often resolve within a month, although they can develop into Stevens–Johnson syndrome. As early as 6 weeks into treatment, hepatitis, especially fulminant hepatitis that results in liver failure, may occur. NNRTIs are not strictly prohibited during pregnancy, although they can interact with hepatic cytochrome P450 enzymes, produce neural tube anomalies, and extend the QT interval [42].

Vivid dreams, delusions, headaches, dizziness, increased suicidality, psychotic-like behavior, and mania are among the mental and CNS side effects that may result from efavirenz [43]. Hepatotoxicity, insulin resistance, hyperglycemia, hyperlipidemia, lipodystrophy, and PR interval lengthening are all linked to the PI class, which includes indinavir and saquinavir. However, because of their ineffectiveness and resistance, other PIs such as indinavir and saquinavir are no longer in use [43]. Due to their neutral effect on triglyceride and cholesterol levels, INSTIs are well tolerated and frequently used as a third therapy in HAART regimens; nonetheless, they may cause adverse effects such as depression and sleeplessness. Dolutegravir can lower GFR, limit creatinine secretion, and interact with antiepileptic drugs, metformin, and rifampin [44, 45]. Although maraviroc is usually well tolerated, certain individuals may develop hepatotoxicity, skin rashes, and disorientation (Table 1) [46, 47].

## 4. Emerging HIV Therapies

### 4.1. Long-Acting Retroviral Drugs

LA-ART is a novel therapeutic approach for HIV prevention and treatment that offers an alternative to daily oral medication for HIV-1 [48]. The five LA-ARTs currently available in a few countries are RPV, ibalizumab, cabotegravir (CAB), lenacapavir, and dapivirine [49]. For the treatment of HIV-1, two LA-ARTs have been approved: Cabenuva, which consists of intramuscular (IM) injections of a combination of LA-CAB and LA-RPV for virologically repressed adolescents and adults averaging nothing less than 35 kg [50]. Based on several clinical trials, including the Phase IIb LATTE-2 study and the Phase III/IIb FLAIR and ATLAS trials, Cabenuva was licensed by Health Canada in March 2020, the European Medicines Agency (EMA) in October 2020, and the US FDA in January 2021 [51]. CAB aqueous nanosuspension, also known as LA-CAB, is stabilized by mannitol, polysorbate 20, and polyethylene glycol 3350. It has a median particle dimension of 200 nm and affects the dissolution profile of the medication depot at the site of injection. For at least 24 weeks, subjects who received doses of ≥ 200 mg LA-CAB showed plasma CAB concentrations that were higher than the protein-adjusted 90% inhibitory concentration (PA-IC90) [52] LA-RPV is a poloxamer-stabilized aqueous nanosuspension of RPV with a median particle dimension of 200 nm (P338). It was first developed for PrEP, but because NNRTI drug resistance profiles were so prevalent, its use was limited. LA-RPV injection was well tolerated and safe in the HPTN-076 study [53]. Many studies have been conducted to determine the efficacy of LA-RPV and LA-CAB in treating HIV-1. Based on the Phase IIb trial LATTE-2 findings, after 256 weeks, patients who received LA-CAB and RPV injections, both 4 or 8 weeks, demonstrated a significant degree of suppression of the virus, equivalent to that of patients who received the conventional oral triple-drug regimen [54]. According to the Phase III FLAIR study, the Q4W pairing of LA-CAB and RPV achieved viral suppression at concentrations comparable to those of conventional oral ART [55]. The recently completed POLAR, MOCHA, and LATITUDE trials (Table 2) corroborated the safety and efficacy of LA-CAB and RPV Q8W [57, 58].

The only capsid-inhibitor-based ART approved for use in patients with multidrug-resistant (MDR) HIV-1 strains who have undergone intensive treatment is Sunlenca (LA LEN) (formerly GS-6207), which is the second LA-ART. The US FDA authorized Sunlenca (LA LEN) in December 2022, after the European Commission approved it in August 2022. LA LEN is administered biannually as a subcutaneous (SC) injection in conjunction with an ideal background regimen (Table 3) [50]. LEN interacts with the NTD–CTD interface, which is a multistage HIV capsid. Resistance-associated mutations in patients who have never received treatment or have received a lot of treatment are uncommon and do not substantially lower the potency of the drug, making it a first-in-class agent. LEN treatment creates a deformed capsid that can enter new target cells but cannot replicate to produce new virions [61, 62]. Mutations linked to LEN resistance are uncommon and do not affect the efficacy of therapy. The efficacy of LEN is unaffected by changes in Gag cleavage sites or polymorphisms linked to PI resistance. In a blinded, randomized, placebo-controlled study that examined the pharmacokinetic (PK) profiles of LEN in SC and oral injectable mixture prescription regimens, 900 mg of LEN administered as a single SC injection produced plasma concentrations of at least 24 ng/mL for at least 26 weeks (Table 3). However, reports of ISRs have been published [50, 63]. LA-ARTs, such as weekly oral or long-acting parenterally delivered drugs, can be helpful when daily oral medications are impractical or inadequately adhered to by patients. However, these methods have limitations in managing toxicities and preventing drug resistance when exposure to these agents is difficult to reverse [64]. Additionally, the dapivirine vaginal ring (DPV-VR), a platinum-catalyzed silicone ring that contains 25 mg of DPV, a method for preventing LA in women, has been approved by five African countries, including Zambia, Zimbabwe, South Africa, Kenya, and Uganda. The ring is safe, well tolerated, and suitable for postmenopausal and sexually active women. The ring was manufactured by the International Partnership for Microbicides (IPM), and the EMA approved it in July 2020. In January 2021, the World Health Organization (WHO) recommended DPV-VR as an additional precautionary measure against HIV-1 infection in high-risk women [65].

However, despite the promising benefits of LA-ARTs, challenges exist, such as therapy cost, which is significantly higher than that of daily oral regimens, potentially limiting accessibility, especially in resource-limited settings [66]. Additionally, ISRs such as nodules, pain, induration, and erythema have been frequently reported, with some cases leading to therapy discontinuation [67]. There is also a risk of drug resistance development in cases of incomplete suppression, particularly if a scheduled injection is missed because of the long PK tail of these formulations [48]. Moreover, the infrastructure required for regular IM or SC administration, including cold-chain storage and trained personnel, poses additional barriers to widespread adoption in LMICs [66, 68]. Therefore, while LA-ART presents an innovative and potentially adherence-improving option, a balanced approach that considers these challenges is critical for its successful implementation.

### 4.2. bNAbs

The recent discovery of potent bNAbs specific to HIV-1 has opened up a new avenue for HIV-1 infection prevention, treatment, and potentially even cure. In the 1990s, first-generation bNAbs were isolated via phage display and human hybridoma electrofusion, with less-than-perfect outcomes (Table 4) and [69, 71]. High-throughput neutralization assays and single-cell antibody cloning techniques have facilitated the isolation and characterization of a new generation of bNAbs with greater potency and range for HIV-1 immunological prophylaxis and treatment. Both methods have been used to identify several bNAbs and novel HIV-1 spike sites that are vulnerable to these neutralizing antibodies [71]. Compared to previous bNAbs, these novel agents showed more than a twofold improvement in coverage and a 10- to 100-fold increase in efficacy [69]. bNAbs are being actively sought after and produced because of their significant advantages, including a longer half-life, superior safety, and activation of the host immune response [72]. Numerous studies have documented functions, including the elimination of free viruses, the removal of infected cells, and the prevention of HIV-1 spread from cell to cell. Furthermore, the rising popularity of bNAbs offers a fresh perspective on vaccine development and encourages immunogen testing [69]. These innovative medications have shown encouraging in vivo effects for both prevention and treatment, in addition to their strong in vitro efficacy. According to studies on rhesus macaques, passive bNAb therapy can protect against high-dose viral challenges at significantly lower blood concentrations or repeated low-dose challenges [73, 74]. Proviral DNA in the peripheral blood, gastrointestinal mucosa, and lymph nodes decreased, and plasma viral RNA quickly decreased to undetectable levels when bNAb was administered to animals in immunotherapy trials with ongoing infections. Furthermore, monoclonal antibody treatment enhances host Gag-specific T-cell responses [75]. However, challenges such as cost are a major concern, as manufacturing and scaling up of monoclonal antibody production are expensive, potentially limiting accessibility in resource-constrained settings. Some bNAbs require intravenous or SC administration, which can cause ISRs, affecting patient acceptability and compliance [76]. The emergence of resistant viral strains, such as HIV-1, necessitates the use of bNAb combinations or bi-/trispecific antibodies, increasing their complexity and cost. The duration of efficacy and frequency of administration are also under investigation, posing logistical challenges for long-term treatment and prevention [77]. Concerns persist regarding the durability and long-term applicability of bNAbs, as they do not lead to durable remission, and their antiviral effects tend to wane over time unless re-administered. This raises questions about their practicality for chronic management, and the need for repeated dosing, potential immunogenicity, and declining neutralization breadth in evolving viral quasispecies complicate their long-term use in diverse patient populations [72].

### 4.3. Gene Editing and CRISPR-Based Therapies

Alternative techniques, such as gene editing, have shown promising outcomes in addition to antiviral therapies and may lead to successful HIV therapy by blocking incorporated HIV DNA [78, 79]. CRISPR–Cas is a recent intriguing gene-editing technique that was first employed in prokaryotes as an immune system response mechanism to fight against bacteriophage infections and invasive plasmids [80–82]. Depending on the type of CRISPR–Cas system, a nuclease called Cas attaches itself to a short CRISPR RNA (crRNA) that binds to complementary viral RNA or DNA sequences. Cas13 is an enzyme that cleaves RNA, whereas Cas9 and Cas12 are endonucleases that cleave DNA. Therefore, integrated proviral HIV DNA can be mutated or removed using CRISPR–Cas-based techniques, which can also be used indirectly to prevent viral receptors from penetrating the cells (Table 4) [70, 83, 84]. Homology-directed repair (HDR) pathways are commonly used [85]. The donor templates contain the required modifications and are encircled by DNA segments that resemble the blunt ends of cleaved DNA. Consequently, a target cell's own HDR DNA repair system can be used to precisely alter its genome. Additionally, Cas13 endonucleases destroy a particular target RNA without modifying the genome and have been employed to prevent HIV replication [86]. The development of a viable curative drug still faces many challenges because of off-targets, immunogenicity, viral escape, and efficient patient administration, notwithstanding the promise that CRISPR–Cas gene editing has demonstrated in HIV treatment [59]. CRISPR–Cas gene-editing therapies face significant implementation challenges in real-world clinical settings because of the need for advanced infrastructure, skilled personnel, and controlled laboratory conditions, despite their promise. Delivery methods for gene-editing components require refinement to ensure safety, efficiency, and tissue-specific targeting [87]. The ethical and regulatory frameworks governing human genome editing are still evolving, potentially delaying clinical adoption. Cost remains a critical barrier, as the individualized nature of gene-editing therapies may restrict accessibility, especially in LMICs disproportionately affected by HIV [80, 88]. New insights into the biology of HIV infection may contribute to the development of CRISPR–Cas-based gene therapy methods [59]. Combination strategies that simultaneously inhibit the viral genome and improve the activity of host-limiting mechanisms are suggested for an infected person to have long-lasting antiviral benefits [89].

### 4.4. Therapies Targeting HIV Latency and Eradication

The shock-and-kill technique is employed to reawaken and eliminate latently infected cells. Latent cell reactivation is accomplished using therapies known as latency reversal agents (LRAs), which remove dormant cells via immune-mediated clearance or virus-mediated cytolysis [90]. Various LRAs can alter chromatin, stimulate transcription, and trigger transcription. In 2020, N-803, an interleukin-15 superagonist, was able to awaken latently infected cells in mice and monkey models; nevertheless, the study was considered unsafe for clinical trials because of the level of damage [91]. The RIVER investigation found no difference in replication-competent proviruses between PLHIV receiving ART or shock-and-kill therapy [92]. Vorinostat (shock) and viral vector vaccination (death) were used to target latent cells in the study; however, the clinical trial failed to show latency reversal or ART stoppage to assess the viral rebound [93]. The shock-and-kill strategy is generally nonspecific. Present-day LRAs frequently result in the global activation of both infected and uninfected T cells. Furthermore, it is challenging to eradicate infected cells after they become active, necessitating improvements to make this shock-and-kill technique effective [94]. The block-and-lock strategy employs several tactics to permanently inhibit the HIV provirus; however, the majority of these strategies only temporarily stop HIV transcription. These pathways are activated by several drugs that limit viral rebound, including didehydro-cortistatin A, LEDGINs, curaxin CBL0100, HSP90 inhibitors, Jak-STAT inhibitors, and ZL058 Tat inhibitors [95]. These methods may alter other biological processes and are not unique to HIV proviral DNA extraction. To epigenetically reduce HIV transcription through histone deacetylation, researchers have employed small interfering RNAs (siRNAs) to target specific areas of the HIV LTR [95, 96].

### 4.5. Nanotechnology-Based Therapies

Viral reservoirs, particularly latently infected cells, pose a significant challenge to the development of an HIV cure. However, nanotechnology offers a promising approach to overcome this obstacle [97]. Nanocarrier drug delivery systems can target these reservoirs, enhance drug delivery, and reduce toxicity. The key benefits of nanocarrier systems for HIV treatment include targeted delivery to specific cells and tissues, increased bioavailability, reduced drug resistance, minimal side effects, and prolonged therapeutic effects [98]. Furthermore, nanocarriers, such as liposomes, dendrimers, and polymeric nanoparticles, have shown potential in overcoming the limitations of traditional HIV treatments. For example, liposomal formulations of drugs, such as zidovudine, improve drug distribution and reduce toxicity [99].

## 5. Breakthrough Strategies in HIV Vaccine Development

Effective HIV vaccine development has been difficult because of HIV diversity, aggressive virulence, and the virus's capacity to elude host immune responses. Five broad categories of vaccine candidates have been identified to date. These include adenovirus vector-based, cytomegalovirus (CMV) vector-based, structural gene-based, and combined envelope–based and structural gene–based vaccines [100]. Most HIV vaccine candidates are subunit proteins, whole inactivated, live attenuated, live vectors, or DNA vaccines [101]. The AIDSVAX gp120 subunit vaccine was the first HIV vaccine developed but was ineffective against HIV during the trial and spurred scientists to consider other vaccine types or approaches in the early 1990s [101, 102]. However, DNA vaccines have emerged as a safe and promising strategy for HIV vaccine development. These vaccines, developed using partial HIV genes, have been proven safe and unlikely to cause infection, providing reassurance and confidence in their potential. Furthermore, recombinant vector vaccines integrate pathogenic genes into harmless or attenuated viruses to elicit immune responses without causing infections or diseases [103]. Therefore, they are being explored as vaccine candidates for HIV infection treatment. Additionally, poxviruses, such as modified vaccinia ankara (MVA), and alphaviruses, such as the Venezuelan equine encephalitis (VEE) virus, are under investigation. A recent research and development landscape study of global vaccines for infectious diseases listed 966 vaccine candidates, with HIV vaccines accounting for 9% of the total [104]. The findings of this study underscore the ongoing efforts to produce a reliable and efficient HIV vaccine, with new vaccine candidates in the pipeline and potential breakthroughs that could change the landscape of HIV prevention. However, HIV vaccine development faces significant challenges owing to the genetic variability of HIV-1, which complicates the design of immunogens for bNAb responses. HIV's ability to establish latent reservoirs early in the infection limits vaccine-induced immune clearance [15, 105]. Eliciting robust and durable immune responses is challenging because traditional vaccine strategies often fail to induce the precise types of immunity required. There is a lack of reliable immune correlates of protection, making it difficult to predict the effectiveness of a candidate vaccine without full-scale efficacy trials [106]. Logistics and ethical challenges, such as participant recruitment, retention, informed consent, and long-term follow-up, also pose challenges, while funding constraints and the high cost of multiphase trials remain persistent, especially in the absence of commercial incentives [107].

Historically, efficacy trials have been completed for several HIV vaccine candidates, each potentially impacting the fight against HIV [108]. The earliest efficacy trials included recombinant gp120-based AIDSVAX vaccine candidates, VAX003 and VAX004, developed to elicit neutralizing antibody responses (nAbs), STEP (HVTN 502), Phambili (HTVN 503), RV144, and HTVN 505 vector-based vaccines aimed at eliciting HIV-specific T-cell responses [93]. However, none of these early efficacy trial candidates were successful in preventing HIV or improving associated parameters (viral load, CD4+, etc.), leading to follow-up trials such as RV305, RV306, RV328, HVTN 097, HVTN 100, HVTN 702 (Uhambo), and HTVN 111 that show scientific resilience and render valuable lessons for future efforts [109]. Recently, the HTVN705 (Imbokodo) and HVTN706 (Mosaico) trials were completed, and the vaccines were reported to be safe, providing reassurance regarding the safety of HIV vaccine development, even if they were not efficacious in preventing HIV-1 infection [110]. Other recent vaccine candidates, including DNA and viral vector vaccines such as Ad4HIV, HVTN119, HVTN124, HIVCORE0052, HIVCORE0051, HIVCORE006, 19-I-0069, and VIR-1111-2001; Env trimer protein-based vaccines such as HVTN122, 19-I-0031, HVTN137, and ACTHIVE-001; and epitope- and lineage-targeting vaccines such as IAVI G001, HVTN133, IAVI C101, HVTN135, and HVTN115, have been developed based on the results of previous trials. However, success has yet to be recorded for human use [109].

## 6. Novel Approaches and Challenges to HIV Vaccine Design

Previous clinical trials have resulted in the development of new HIV vaccine design approaches that focus on immunogens and adjuvants. These strategies can boost immune responses and elicit the production of neutralizing antibodies against the virus. New immunogens increase the number of epitopes for bNAbs, overcome immunodominance challenges, and demonstrate a strong affinity for diverse antigenic sites, thereby enhancing vaccine effectiveness [108]. Due to the decline in vaccine efficacy in earlier clinical studies, researchers are investigating novel adjuvants to improve protein vaccine responses. Four adjuvants have enhanced immune responses and offer important insights into the development of future vaccines: liposomes, oil-in-water emulsions, and aluminum salts containing MPLA [109]. Other novel vaccine approaches, including Ad26 mosaic trials, the PrEPVacc trial, the utilization of new viral vectors such as CMV, and antibody-mediated protection (AMP) trials, have been described by Kim et al. [109]. It is important to note that although artificial intelligence (AI) does not replace traditional HIV vaccine development methods, it plays a supportive and increasingly valuable role. AI has the potential to accelerate progress by addressing key challenges in antigen selection, immunogen design, adjuvant discovery, and epitope prediction [111, 112]. It also complements conventional scientific methods by reducing costs and time and optimizing the results. Using approaches such as big data, machine learning (ML), and generative models, AI improves the reliability and efficiency of HIV vaccine candidates and accelerates efforts toward a successful HIV vaccine [113]. However, to fully utilize the benefits of AI integration in vaccine production, challenges such as limited access to high-quality, diverse datasets, model interpretability, and alignment with heterogeneous global regulatory frameworks must be addressed [114].

Despite efforts to develop a safe HIV-1 vaccine, the epidemiology and pathogenesis of this virus pose significant social and scientific challenges to vaccine development. Genetic diversity, limitations of animal models in disease studies and vaccine trials, and depletion of effector cells (CD4+ and monocytes) at early infection stages are the primary scientific challenges hindering the development of an effective HIV vaccine [114]. HIV's antigenic diversity complicates immunogen design, making it difficult to create vaccines that induce bNAbs against all circulating variants. The early establishment of latent reservoirs and integration of the virus into the host genome also challenge vaccine-induced sterilizing immunity [15]. The lack of reliable immune correlates of protection further hinders progress, necessitating large-scale and complex clinical trials. Additionally, limitations in animal models prevent accurate prediction of human immune responses, as nonhuman primate studies do not fully replicate HIV pathogenesis in humans, making translational progress uncertain [115]. Social challenges, such as multiple transmission routes for the virus due to a variety of lifestyle factors, willingness, retention, and sexual risks associated with people participating in efficacy trials, and issues surrounding ethics and animal welfare, have been identified in HIV vaccine development [116]. HIV is a rapidly mutating RNA retrovirus that presents a scientific challenge because of its rapid replication and recombination cycle. The rapid development of multiple viral variants within infected individuals leads to unique immune evasion against vaccine candidates [117]. The difficulty in identifying and maintaining sensitive animal models and advocating animal welfare further complicates vaccine development. HIV's ability to destroy effector T cells in early infection emphasizes the need for vaccine-induced immunity, further complicating the potency of the immune response [114]. Large-scale vaccine trials, particularly in resource-limited HIV-prone areas, present infrastructural and ethical challenges, including maintaining participant engagement and ensuring culturally sensitive recruitment and informed consent [118].

## 7. Advancements in HIV Cure and Prevention Strategies

Only three cases of HIV cure have been reported in more than 40 years since the first instance of AIDS was discovered, including the London and Berlin patients. Patients in London and Berlin received bone marrow transplants as part of a complex, costly, and time-consuming strategy that was unfeasible for 38 million PLHIV. However, HIV infection is now viewed as a chronic condition that may be well controlled rather than a fatal illness [50]. There have long been two types of HIV cures under development: sterilizing and functional cures. Sterilizing cures involve the complete removal of replication-competent proviruses, whereas functional cures involve long-term control of HIV replication, including maintaining a normal CD4 T-cell count and HIV replication below detectable levels [119]. Sterilizing cures have only been documented in one instance, involving the well-known “Berlin patient,” Timothy Brown, who developed HIV-1 resistance after receiving two stem cell transplants from a donor homozygous for the CCR5delta32 mutation, a CCR-deficient treatment for acute myeloid leukemia (AML) [120]. Due to the challenge of locating donors whose human leukocyte antigens (HLA) match those of recipients for CCR5 Delta32/Delta32 stem cell transplantation, this method of sterilizing a cure is not replicable for infectious diseases such as HIV. The most promising therapeutic techniques for attaining a sterilizing cure for HIV are gene-based medicines and the use of latency-reversing drugs based on “shock-and-kill” strategies [119].

One important tactic for attaining a functional cure for HIV is ART. Elite controllers are a tiny percentage of HIV-positive people who can permanently stop HIV replication without the aid of ART or any other kind of treatment [121]. The goal of a functional cure is to achieve a state of host-mediated viral replication control in which, in the absence of ART, the immune function is stabilized and restored, HIV-1-induced inflammation is decreased, and the plasma viral load is maintained at extremely low levels, thereby lowering the risk of virus transmission. This suggests that patients can safely stop using ART because their immune system can regulate viral replication [122]. A combination of immune-based therapies, especially those involving bNAbs and vaccine-based therapies, has been the most explored strategy for achieving a functional cure for HIV [119]. Key HIV prevention measures that target people at high risk of infection include pre-exposure prophylaxis (PrEP) and postexposure prophylaxis (PEP). To protect those who are often exposed to HIV, including those who have several partners by choice or occupation, PrEP, which is taken before exposure, entails the regular use of a combination of ART such as emtricitabine/tenofovir. Its effectiveness depends on adherence, and frequent monitoring is necessary to control possible drug-related negative effects [123, 124]. In contrast, PEP is initiated immediately after a known exposure to HIV and is most effective when taken promptly, as its efficacy decreases with delayed administration [123]. These interventions bridge critical gaps in prevention efforts and strengthen the fight against HIV transmission among vulnerable populations [125].

To address compliance issues with daily pills associated with PrEP, long-acting injectable LAI-PrEP and the use of vaginal microbicides by women are options being explored. The LAI-PrEP addresses adherence issues related to oral PrEP regimens. For example, IM administration of CAB every 8 weeks is more efficacious than oral formulations such as tenofovir disoproxil fumarate and emtricitabine [107]. Other LAI-PrEPs, such as islatravir, with the potential for nonerodable SC implants that can deliver prophylaxis for up to 12 months, have been developed and tested with good efficacy results but are currently undergoing investigation due to safety concerns that led to their discontinuation for human use in 2022 [108]. Vaginal microbicides are chemical substances that are introduced into the vagina and, occasionally, the rectum to avoid sexual transmission of HIV and other transmissible diseases, including herpes. Several of these products aim to strengthen the vagina's natural defenses to inactivate HIV and prevent its fusion to host cells and replication in the genital tract [109]. Despite its promise, efficacy trials have yet to prove an effective vaginal microbicide candidate for HIV prevention or PrEP [123].

## 8. Challenges to the Progress

**Figure 1:**
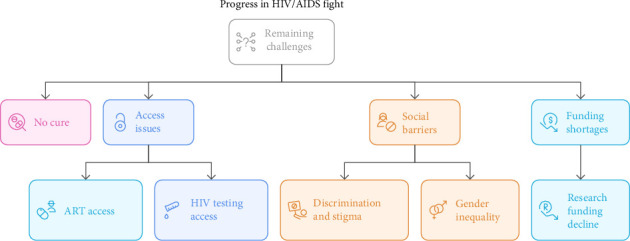
Challenges to progress in the fight against HIV/AIDS.

Over the past 20 years, efforts have been made to combat HIV and significant progress has been made. This is highlighted by the reduced number of AIDS-related deaths and new infections, particularly in the pediatric population. However, control efforts have been hampered by these lingering issues (Figure 1). There is still no cure for HIV, and many individuals who are infected or at risk do not have access to care, prevention, or treatment. Food insecurity, other infectious diseases, and general global health and development concerns are among the other difficulties faced by nations most affected by HIV [126, 127]. Access to HIV testing and ART is one of the most crucial concerns in the global battle against HIV. Significant obstacles remain in guaranteeing fair access to testing and treatment, despite initiatives to increase awareness and upgrade healthcare facilities. Approximately 9.2 million PLHIV failed to receive treatment in 2022, and 5.5 million were unaware of their infection [128]. Discrimination and stigma are significant challenges in the global battle against HIV/AIDS. Individuals and communities are severely impacted by unfavorable social perceptions of those who are HIV/AIDS-positive. This discourages people from being tested, disclosing their status, and adopting safer practices [129]. Additionally, gender inequality contributes to the increased susceptibility to HIV infection among women and girls. Women's access to healthcare and safe sex is restricted by societal standards, unequal power relations, and violence [130]. For example, by 2022, women and girls accounted for 63% of all newly diagnosed HIV infections in the WHO African region [128]. Moreover, inadequate funding for HIV/AIDS research and treatment is a significant drawback [128, 131]. A decrease in research funding has resulted in fewer scientific discoveries, slowing the development of new ART and preventive strategies. As a result, creating an AIDS-free world is delayed, and innovation is stifled [127].

## 9. Recommendations

**Figure 2:**
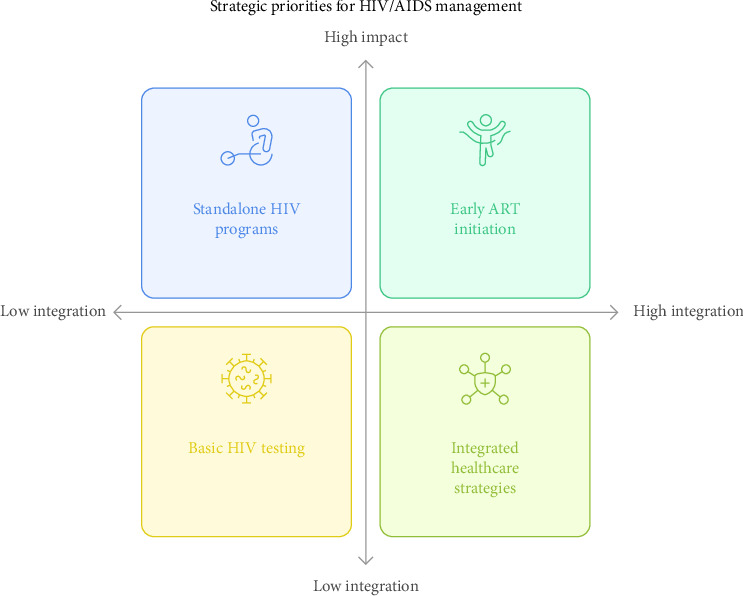
Strategic priorities for HIV/AIDS management.

To stimulate Global Fund HIV investments in under-resourced settings, core and noncore funds from several partners and advancing a multisectoral and inclusive global response to end AIDS by 2030 are expedient [132]. Reducing infections, enhancing the quality of life for individuals living with HIV, and eventually putting an end to the epidemic all depend on a comprehensive strategy that addresses the structural, societal, and personal causes of the HIV/AIDS pandemic. This approach should integrate science, technology, and community engagement [127]. Increasing HIV testing uptake is a key priority, as testing is the entry point in the HIV care cascade and is essential for epidemic control (Figure 2). The UNAIDS aims to ensure that 95% of individuals living with HIV are aware of their status by 2025, emphasizing the importance of accessible and accurate testing [133, 134]. Promptly connecting individuals who test positive to care enhances health outcomes, reduces mortality, and improves overall well-being. In addition to improving personal health, early ART commencement dramatically lowers the likelihood of HIV transmission. Early ART reduces the spread of HIV to healthy partners by 93% [133].

Integrating standalone HIV programs into primary healthcare platforms is crucial for ending the HIV epidemic and promoting population health and sustainability. This approach has improved health outcomes in sub-Saharan Africa (SSA) and supported the achievement of the 2030 AIDS eradication target [135, 136]. Access to care for hard-to-reach populations has increased, ART adherence has improved, and HIV stigma has decreased through noncommunicable disease (NCD) and coordinated HIV services. These programs also lower the costs of HIV and non-HIV care [136–139]. Mental health integration enhances ART adherence, particularly when treating depression and HIV infection. In high-income countries, depression treatment increased ART adherence by 83%, whereas interventions such as cognitive behavioral therapy and alcohol screening have shown similar success in LMICs [140–143]. Additionally, integrated healthcare strategies improve outcomes for chronic conditions such as hypertension, asthma, diabetes, and epilepsy. For example, integrated chronic care clinics in rural Malawi have yielded better clinical results for HIV and other chronic diseases [144]. In South Africa, integrating ART into maternity and child health programs improved viral suppression and breastfeeding outcomes for women living with HIV compared with standalone services [145]. These findings highlight the benefits of integrating HIV care into broader health services; however, further research is required to refine these models. Studies should assess the clinical outcomes, service delivery metrics, and sustainability factors, such as financing, human resources, and local conditions [135].

HIV treatment and research have progressed significantly through collaboration between governments, pharmaceutical companies, and nongovernmental organizations. These partnerships have led to the development of life-saving antiretroviral drugs, improved HIV prevention techniques, and a reduction in mortality rates. However, challenges remain in ensuring equitable access, particularly in resource-limited settings [146]. Global health frameworks, such as the Sustainable Development Goals (SDGs) and UNAIDS, emphasize multisector collaboration to achieve universal health coverage and end the AIDS epidemic by 2030. Pharmaceutical companies drive research, governments provide funding and policy guidance, and NGOs ensure that interventions reach vulnerable populations [4]. Funding bodies such as the Bill & Melinda Gates Foundation, NIH, and the Global Fund are essential for advancing HIV research. These organizations provide financial research support, encourage international collaboration, and shape research priorities in the field. For example, the NIH funds initiatives such as the NIH AIDS Clinical Trials Group (ACTG) to discover novel therapies and vaccines [147]. They also support efforts to improve access to ART for marginalized populations and to develop affordable vaccines [148]. Additionally, funding organizations work to build research capacity in areas where HIV is most prevalent. In addition, the NIH Fogarty International Center empowers local scientists by funding HIV-related workshops in the SSA [149]. They also promote the unrestricted sharing of research findings, as seen in the European and Developing Countries Clinical Trials Partnership (EDCTP) [150].

To promote the widespread use of emerging HIV therapies in LMICs, governments and international stakeholders should prioritize technology transfer agreements, support local production of LA-ART, and incentivize pharmaceutical companies to adopt tiered pricing models. Simplified supply chains, decentralized service delivery, and capacity building for local health workers can ensure the sustainability of these innovations [151]. Policy changes should integrate LA-ART and PrEP into the national HIV treatment guidelines, with clear implementation frameworks, financing mechanisms, and performance metrics. Addressing stigma, access barriers, and structural challenges requires community-led advocacy, cultural competence training, and the creation of supportive environments. Legal and policy reforms to decriminalize HIV transmission, sex work, and drug use are crucial [152]. Moreover, future research should focus on exploiting computational tools to address the critical stages of the HIV life cycle, including entry, reverse transcription, integration, assembly, and release. Targeting key viral proteins, such as integrase, protease, and reverse transcriptase, along with co-receptors involved in viral entry, represents a promising avenue for therapeutic innovation [153]. Optimizing medication design and increasing predictive accuracy are two significant benefits of integrating cutting-edge innovations, such as AI and ML. Identifying novel therapeutic targets and refining treatment strategies could significantly enhance the effectiveness of HIV management, ultimately contributing to better patient outcomes and global efforts to combat the disease [154].

## 10. Conclusion

Over the last 4 decades, HIV research has made significant progress in understanding HIV immunology, leading to the development of potent ART. However, cART cannot completely eradicate the virus and is associated with long-term health complications. Innovative therapeutic approaches, such as latency-reversing therapies, block-and-lock strategies, gene-editing technologies, and immune-based interventions, are cost-effective and promising. Vaccine development remains a high-priority area but faces obstacles due to HIV's rapid mutation rate and viral envelope variability. Therefore, continued innovation and global collaboration are crucial for addressing these challenges and achieving long-term control and eradication of HIV.

## Figures and Tables

**Table 1:** FDA-approved HIV antiretroviral drugs by class and approval date [25–27].

Drug class	Drug	Approval date
Non-nucleoside reverse transcriptase inhibitors (NNRTIs)	Etravirine	Jan 2008
	Delavirdine	June 1997
	Efavirenz	Sept 1998
	Nevirapine	June 1996

Nucleoside reverse transcriptase inhibitors (NRTIs)	Abacavir	Dec 1998
	Didanosine	Oct 1991
	Emtricitabine	July 2003
	Lamivudine	Nov 1995
	Stavudine	June 1994
	Zalcitabine	June 1992
	Zidovudine	March 1987
	Tenofovir disoproxil fumarate	Oct 2021

Integrase inhibitors	Cabotegravir	Jan 2021
	Dolutegravir	Aug 2013
	Raltegravir	Oct 2007

Protease inhibitors (PIs)	Atazanavir	June 2003
	Darunavir	June 2006
	Fosamprenavir	Oct 2003
	Ritonavir	March 1996
	Tipranavir	June 2005

Entry inhibitors	Maraviroc	Aug 2007
	Fostemsavir	July 2020
	Ibalizumab-uiyk	March 2016

Fusion inhibitors	Enfuvirtide	March 2003

**Table 2:** Comparative analysis of clinical trials evaluating long-acting cabotegravir (CAB) and rilpivirine (RPV) regimens for HIV treatment and maintenance [50, 54, 56].

	LATTE-2	FLAIR	ATLAS	ATLAS-2M
Aim	Safety and effectiveness of the LA-CAB and RPV regimen in PLWH who have never received therapy	Assessment of the efficacy and effectiveness of LA-CAB and RPV as maintenance medication after switching from an INSTI plus two NRTI regimens in participants who have never received HIV treatment	Assessment of the noninferiority of LA-CAB and RPV in comparison to continuing the present daily oral treatment in PLWH with viral suppression	Evaluation of the effectiveness of LA-CAB and RPV as sustenance therapies given in 8 weeks instead of in 4 weeks.

Participants	Eligibility: PLWH ≥ 18 years old, on ART for ≤ 10 days, with ≥ 200 mm CD4+ T-cell counts and ≥ 1000 copies/mL of HIV-1 RNA are eligible. There were 286 participants, ages 19–64.	Eligibility: HIV-1 RNA plasma levels > 1000 copies/mL and treatment-naïve PLWH ≥ 18 years of age. There were 566 participants (18–68 years old), and 127 of whom were female (22%).	Eligibility: HIV-positive individuals aged 18 and older who are on continuous antiretroviral therapy (ART) without CVF. Seventy-four percent of the 618 participants, ages 18 to 82, had CD4+ T-cell levels of 500/mL^3^.	Eligibility: First or second oral standard-of-care regimen for ≥ 6 months for virally suppressed PLWH. Participants: 1045 individuals aged 34–50.

Study design	Oral CAB + ABC/3TC was administered to participants for 20 weeks, with RPV added 4 weeks before randomization. Groups 2 and 3 got LA-CAB + RPV every 4 or 8 weeks, while Group 1 continued oral treatment. Participants either switched to or maintained Q8W/Q4W LA treatment in Week 256.	After a 4-week oral lead-in (CAB 30 mg, RPV 25 mg) and a loading dosage (600 mg/900 mg), participants were randomly assigned to either (1) continue oral treatment or (2) transition to LA-CAB + RPV (400 mg/600 mg Q4W) following 16 weeks of daily oral DTG + ABC + 3TC (50 mg/600 mg/300 mg) for viral suppression. Participants in oral groups had the option to withdraw at week 100 or switch to LA treatment with or without a 4-week oral lead-in.	Participants were divided into two groups: Gr-1 (oral therapy) and Gr-2 (transition to LA therapy). Gr-2 received an oral lead-in for 4 weeks, followed by intramuscular injections and injections every 4 weeks. After week 52, participants had options to switch to LA therapy, withdraw, transfer to ATLAS-2M, or continue LA therapy during the study extension phase.	Every 8 weeks (Q8W), participants were randomly assigned to one of two groups: 600 mg of LA-CAB with 900 mg of LA-RPV is the dosage for Gr-1; 400 mg of LA-CAB plus 600 mg of LA-RPV is the dosage for Gr-2, which is administered in 4 weeks (Q4W). Those who did not receive CAB/RPV were given a single-daily OLI for 4 weeks (30 mg of CAB + 25 mg of RPV).

Results	Viral suppression rates were 84% (oral group), 87% (Q4W group), and 94% (Q8W group) at Week 96. Two (Q8W) and one (oral group) patients had CVF. Mild (84%) and moderate (15%) ISRs were the most common. With sporadic discontinuations brought on by CVF or ISR, virus suppression was 81% for the Q4W/Q8W groups and 93% for the extension-switch group at Week 256.	Both the LA (93.6%) and oral treatment (93.3%) groups had strong virological suppression rates at week 48, with 86% of LA group patients reporting mild-to-moderate ISRs. Three percent of individuals in each group had HIV-1 RNA > 50 copies/mL at Week 96, with an AE incidence of 8% that was similar. The percentage of individuals with HIV-1 RNA > 50 copies/mL rose to 5% at Week 124, including five new cases since Week 96, whereas 99% (110/111) of direct-to-injection and 93% (113/122) of OLI group members were still virally suppressed.	92.5% (288/311) of the LA group and 95.5% (286/300) of the oral treatment group were still virally suppressed at week 48; three and four subjects, respectively, had been reported to have CVF. The LA group's most frequent adverse events were ISRs. All 23 patients in the LA arm were still virologically suppressed at Week 96, whereas one person (*n* = 29) in the switch arm had HIV-1 RNA > 50 copies/mL. There were no documented incidences of CVF in either group.	By Week 48, the Q8W and Q4W arms had viral suppression rates of 94.3% and 93.5%, respectively, while 1.5% (8/522) and 0.4% (2/523) of subjects had CVF. With a total of 9 CVFs in Q8W and 2 in Q4W, including one in Q8W at Week 48, HIV-1 RNA > 50 copies/mL was recorded in 2% (Q8W) and 1% (Q4W) by Week 96. The two other CVFs happened between weeks 96 and 152.

Comments	Whether given every four or 8 weeks, LA treatment (LA-CAB and RPV) was just as effective at sustaining viral suppression as daily three-drug oral therapy.	The effectiveness of oral treatment with DTG/3TC/ABC and LA therapy (CAB and RPV) in sustaining viral suppression was comparable.	In virally suppressed PLWH, LA treatment (LA-CAB and RPV) was as effective as continuing the existing oral medication.	Administering LA-CAB and RPV every 8 weeks was just as effective as administering them every 4 weeks.

Abbreviations: AEs = adverse events, CVF = confirmed virological failure, IM = intramuscular; ISRs = injection site reactions, OLI = oral lead-in, PLWH = people living with HIV, Q4Q = once every 4-week injection, Q8W = once every 8-week injection.

**Table 3:** Summary of clinical trials evaluating lenacapavir (LEN) for HIV treatment [50, 59, 60].

Clinical trial	CAPELLA (NCT04150068)	CALIBRATE (NCT04143594)
Aims	Evaluation of LEN's efficacy and safety in conjunction with the optimal baseline treatment in patients infected with MDR HIV-1.	Evaluation of LA LEN's efficacy and safety in combination with additional ARTs among PLWH.
Participants	Eligibility: Individuals aged 12 years or older, weighing 35 kg or more, who were on ART for more than 8 weeks, had an MDR infection, and had ≥ 400 copies/mL of HIV-1 RNA. Participants: 72 people, ages 23 to 78. 25 percent are women. VL was 4.17 ± 1.03 × log10 copies/mL on average.	Eligibility: Participants who are naïve to treatment and have > 200 copies/mL of HIV-1 RNA are at least 18 years old. 182 people participated, with 7% of them being female.
Study design	The study involved 36 participants divided into two groups. Cohort 1 received LEN or a placebo orally for 14 days, followed by 927 mg LEN SC injections every 6 months. Cohort 2 received LEN injections every 6 months after 14 days of oral LEN treatment. Participants were given an ideal background routine.	The study involved four randomly divided participants who received LEN SC injections every 6 months, along with FTC/TAF. Group 1 continued LEN injections until week 28, while Group 2 switched to SC injections in addition to oral BIC. Group 3 received LEN + FTC/TAF daily, while Group 4 received BIC/FTC/TAF. The study aimed to understand the effects of different treatment regimens.
Virological efficacy and adverse events	The study found that 88% of participants in the oral LEN group experienced a reduction in HIV-1 RNA from baseline within 14 days, while 17% of the control group did not. After 26 weeks, 81% and 83% of patients experienced viral suppression, respectively. Two subjects experienced breakthrough capsid mutations resistant to LEN.	At Week 28, the viral suppression rates for cohorts 1, 2, 3, and 4 were 94%, 92%, 94%, and 100%, respectively. Cohorts 1, 2, 3, and 4 had suppressed viral rates of 90%, 85%, 85%, and 92% at Week 54, respectively. No severe adverse events were noted. However, three subjects suffered Grade-1 ISRs, which resulted in the cessation of LEN injections.
Comments	LEN therapy greatly enhanced treatment results in MDR patients.	SC or oral LEN injections in combination with BIC, FTC/TAF, or TAF are tolerated effectively and maintain an elevated degree of virological repression for PLWH who are new to therapy.

**Table 4:** Summary of emerging HIV therapies [50, 52, 69, 70].

Therapy type	Description	Notable drugs or strategies	Key trials and findings
Long-acting retroviral drugs (LA-ART)	LA-ART offers an alternative to daily oral HIV medications, addressing adherence challenges.	- Cabenuva: Combines LA-cabotegravir (CAB) and LA-rilpivirine (RPV) injections for virologically suppressed patients.	-Cabenuva trials: - LATTE-2, FLAIR, ATLAS: Demonstrated efficacy and viral suppression rates comparable to oral ART.
			- POLAR, MOCHA, LATITUDE: Support Q8W dosing of LA-CAB and RPV.
		- Sunlenca (LA LEN): Capsid inhibitor for MDR HIV-1 strains.	- Sunlenca approval: Approved by the EU commission (August 2022) and US FDA (December 2022).
		- Dapivirine vaginal ring (DPV-VR): Women-focused prevention strategy.	- DPV-VR: Approved in 5 African countries and EMA for HIV prevention in women.

Broadly neutralizing antibodies (bNAbs)	Advanced HIV-1 immunological prevention and treatment using high-potency antibodies.	Novel bNAbs were developed with improved coverage and potency compared to earlier generations. Demonstrated longer half-life, superior safety, and activation of host immune responses.	- Efficacy studies: - Animal models showed protection against viral challenges and reduction in viral RNA.
			- Improved host T-cell responses.
			- Offers insights into vaccine development and immunogen testing.

Gene editing and CRISPR-based therapies	CRISPR–Cas systems can target integrated HIV DNA for mutation or removal, offering a potential functional HIV cure.	- CRISPR–Cas9/Cas12: DNA editing for removing integrated proviral HIV DNA.	- Potential benefits: - Effective targeting of viral DNA/RNA.
			- Prevention of HIV replication using Cas13.
		- CRISPR–Cas13: RNA editing for preventing HIV replication.	- Combined strategies are proposed to enhance antiviral effects.
		- HDR pathway is used for precise genome editing.	- Challenges: - Include delivery, immunogenicity, viral escape, and off-target effects.

## Data Availability

Data sharing is not applicable to this article as no datasets were generated or analyzed during the current study.

## Nomenclature

AIDS: Acquired immune deficiency syndrome
ART: Antiretroviral therapy
CAB/RPV: Cabotegravir/rilpivirine
CAB-LA: Long-acting cabotegravir
CBOs: Community-based organizations
COVID-19: Coronavirus disease 2019
FDA: Food and Drug Administration
HCPs: Healthcare providers
HIV: Human immunodeficiency virus
IDU: Injection drug use or injecting drug user
LA-ART: Long-acting antiretroviral therapy
LMICs: Low- and middle-income countries
MOH: Ministry of Health
MSM: Men who have sex with men
NCDs: Noncommunicable diseases
NGOs: Nongovernmental organizations
NIH: National Institutes of Health
PLHIV: People living with HIV
PrEP: Pre-exposure prophylaxis
QALY: Quality-adjusted life year
RCTs: Randomized controlled trials
SDGs: Sustainable development goals
STIs: Sexually transmitted infections
TDF/FTC: Tenofovir disoproxil fumarate/emtricitabine
UNAIDS: Joint United Nations Programme on HIV/AIDS
WHO: World Health Organization
WHO AFRO: World Health Organization Regional Office for Africa.

## References

[1] Lacombe K., Pacanowski J. (2006). HIV Infection and Comorbidities. *Revue du Praticien*.

[2] Ho Y. C., Shan L., Hosmane N. N. (2013). Replication-Competent Noninduced Proviruses in the Latent Reservoir Increase Barrier to HIV-1 Cure. *Cell*.

[3] De Cock K. M., Jaffe H. W., Curran J. W. (2012). The Evolving Epidemiology of HIV/AIDS. *AIDS*.

[4] Joint United Nations Programme on HIV/AIDS (UNAIDS) (2024). Fact Sheet 2024-Latest Global and Regional HIV Statistics on the Status of the AIDS Epidemic. https://www.unaids.org/en.

[5] Powell R. L. R., Urbanski M. M., Burda S., Kinge T., Nyambi P. N. (2009). High Frequency of HIV-1 Dual Infections Among HIV-Positive Individuals in Cameroon, West Central Africa. *Journal of Acquired Immune Deficiency Syndromes*.

[6] Wensing A. M., Calvez V., Ceccherini-Silberstein F. (2022). 2022 Update of the Drug Resistance Mutations in HIV-1. *Topics in Antiviral Medicine*.

[7] Jena R., Vishwas S., Kumar R. (2022). Treatment Strategies for HIV Infection With Emphasis on Role of CRISPR/Cas9 Gene: Success So Far and Road Ahead. *European Journal of Pharmacology*.

[8] Stephenson K. E. (2018). Therapeutic Vaccination for HIV. *Current Opinion in HIV and AIDS*.

[9] Etemad B., Esmaeilzadeh E., Li J. Z. (2019). Learning from the Exceptions: HIV Remission in Post-Treatment Controllers. *Frontiers in Immunology*.

[10] Chawla A., Wang C., Patton C. (2018). A Review of Long-Term Toxicity of Antiretroviral Treatment Regimens and Implications for an Aging Population. *Infectious Disease and Therapy*.

[11] Gao F., Weaver E. A., Lu Z. (2005). Antigenicity and Immunogenicity of a Synthetic Human Immunodeficiency Virus Type 1 Group M Consensus Envelope Glycoprotein. *Journal of Virology*.

[12] World Health Organization (2015). *Guideline on When to Start Antiretroviral Therapy and on Pre-Exposure Prophylaxis for HIV*.

[13] Foka F. E. T., Mufhandu H. T. (2023). Current ARTs, Virologic Failure, and Implications for AIDS Management: A Systematic Review. *Viruses*.

[14] Schwarzer R., Gramatica A., Greene W. C. (2020). Reduce and Control: A Combinatorial Strategy for Achieving Sustained HIV Remissions in the Absence of Antiretroviral Therapy. *Viruses*.

[15] Boomgarden A. C., Upadhyay C. (2025). Progress and Challenges in HIV-1 Vaccine Research: A Comprehensive Overview. *Vaccines*.

[16] Lanzafame M., Mori G., Vento S. (2025). Advances in HIV Treatment: Long-Acting Antiretrovirals and the Path Toward a Cure. *Biomedicines*.

[17] World Health Organization (2024). Treatment & Care. https://www.who.int/teams/global-hiv-hepatitis-and-stis-programmes/hiv/treatment.

[18] Bekker L. G., Alleyne G., Baral S. (2018). Advancing Global Health and Strengthening the HIV Response in the Era of the Sustainable Development Goals: The International AIDS Society—Lancet Commission. *The Lancet*.

[19] Namasivayam V., Vanangamudi M., Kramer V. G. (2019). The Journey of HIV-1 Non-Nucleoside Reverse Transcriptase Inhibitors (NNRTIs) From Lab to Clinic. *Journal of Medicinal Chemistry*.

[20] Choi J., Horner K. A., Carnevale K. (2024). *Atazanavir*.

[21] Burke L. A., Gulick R. M. (2018). Entry Inhibitors. *Encyclopedia of AIDS*.

[22] Xiao T., Cai Y., Chen B. (2021). HIV-1 Entry and Membrane Fusion Inhibitors. *Viruses*.

[23] Smith S. J., Zhao X. Z., Passos D. O., Lyumkis D., Burke T. R., Hughes S. H. (2021). Integrase Strand Transfer Inhibitors Are Effective Anti-HIV Drugs. *Viruses*.

[24] Lennox J. L., Landovitz R. J., Ribaudo H. J. (2014). Efficacy and Tolerability of 3 Nonnucleoside Reverse Transcriptase Inhibitor-Sparing Antiretroviral Regimens for Treatment-Naive Volunteers Infected With HIV-1. *Annals of Internal Medicine*.

[25] Arts E. J., Hazuda D. J. (2012). HIV-1 Antiretroviral Drug Therapy. *Cold Spring Harbor Perspectives in Medicine*.

[26] Hivinfo.Nih.gov (2020). FDA-Approved HIV Medicines.

[27] International Association of Providers of AIDS Care (2021). How Entry Inhibitors Work.

[28] Ford N., Migone C., Calmy A. (2018). Benefits and Risks of Rapid Initiation of Antiretroviral Therapy. *AIDS*.

[29] Günthard H. F., Saag M. S., Benson C. A. (2016). Antiretroviral Drugs for Treatment and Prevention of HIV Infection in Adults: 2016 Recommendations of the International Antiviral Society-USA Panel. *JAMA*.

[30] Thompson M. A., Aberg J. A., Hoy J. F. (2012). Antiretroviral Treatment of Adult HIV Infection: 2012 Recommendations of the International Antiviral Society-USA Panel. *JAMA*.

[31] Lundgren J. D., Babiker A. G., Gordin F. (2015). Initiation of Antiretroviral Therapy in Early Asymptomatic HIV Infection. *New England Journal of Medicine*.

[32] Paterson D. L., Swindells S., Mohr J. (2000). Adherence to Protease Inhibitor Therapy and Outcomes in Patients With HIV Infection. *Annals of Internal Medicine*.

[33] Sahay S., Dhayarkar S., Reddy K. (2011). Optimizing Adherence to Antiretroviral Therapy. *Indian Journal of Medical Research*.

[34] Abadiga M., Hasen T., Mosisa G., Abdisa E. (2020). Adherence to Antiretroviral Therapy and Associated Factors Among Human Immunodeficiency Virus Positive Patients Accessing Treatment at Nekemte Referral Hospital, West Ethiopia, 2019. *PLoS One*.

[35] Li J. Z., Gallien S., Ribaudo H., Heisey A., Bangsberg D. R., Kuritzkes D. R. (2014). Incomplete Adherence to Antiretroviral Therapy Is Associated With Higher Levels of Residual HIV-1 Viremia. *AIDS*.

[36] Haynes J., Joshi A., Larue R. C., Eisenmann E. D., Govindarajan R. (2024). Nucleoside Reverse Transcriptase Inhibitor (NRTI)-Induced Neuropathy and Mitochondrial Toxicity: Limitations of the Poly-γ Hypothesis and the Potential Roles of Autophagy and Drug Transport. *Pharmaceutics*.

[37] Chen Y. F., Stampley J. E., Irving B. A., Dugas T. R. (2019). Chronic Nucleoside Reverse Transcriptase Inhibitors Disrupt Mitochondrial Homeostasis and Promote Premature Endothelial Senescence. *Toxicological Sciences*.

[38] Last I., Jortner J. (2005). Regular Multicharged Transient Soft Matter in Coulomb Explosion of Heteroclusters. *Proceedings of the National Academy of Sciences of the United States of America*.

[39] Lucas G. M., Ross M. J., Stock P. G. (2014). Clinical Practice Guideline for the Management of Chronic Kidney Disease in Patients Infected With HIV: 2014 Update by the HIV Medicine Association of the Infectious Diseases Society of America. *Clinical Infectious Diseases*.

[40] Lyseng-Williamson K. A., Reynolds N. A., Plosker G. L. (2005). Tenofovir Disoproxil Fumarate. *Drugs*.

[41] Martin A. M., Nolan D., Gaudieri S. (2004). Predisposition to Abacavir Hypersensitivity Conferred by HLA-B∗5701 and a Haplotypic Hsp70-Hom Variant. *Proceedings of the National Academy of Sciences*.

[42] Ye Y., Zhang Q., Tan Y. W. (2024). Toxic Epidermal Necrolysis Caused by Viral Hepatitis A: A Case Report and Literature Review. *Frontiers of Medicine*.

[43] Julie S., Eggleton S. N. (2023). *Highly Active Antiretroviral Therapy (HAART)*.

[44] Hoffmann C., Welz T., Sabranski M. (2017). Higher Rates of Neuropsychiatric Adverse Events Leading to Dolutegravir Discontinuation in Women and Older Patients. *HIV Medicine*.

[45] Zash R., Makhema J., Shapiro R. L. (2018). Neural-Tube Defects With Dolutegravir Treatment From the Time of Conception. *New England Journal of Medicine*.

[46] De Luca A., Pezzotti P., Boucher C. (2019). Clinical Use, Efficacy, and Durability of Maraviroc for Antiretroviral Therapy in Routine Care: A European Survey. *PLoS One*.

[47] Miao M., De Clercq E., Li G. (2020). Clinical Significance of Chemokine Receptor Antagonists. *Expert Opinion on Drug Metabolism & Toxicology*.

[48] Nachega J. B., Scarsi K. K., Gandhi M. (2023). Long-Acting Antiretrovirals and HIV Treatment Adherence. *The Lancet HIV*.

[49] Sherman E. M., Agwu A. L., Ambrosioni J. (2024). Consensus Recommendations for Use of Long‐Acting Antiretroviral Medications in the Treatment and Prevention of HIV‐1: Endorsed by the American Academy of HIV Medicine, American College of Clinical Pharmacy, Canadian HIV and Viral Hepatitis Pharmacists Network, European AIDS Clinical Society, and Society of Infectious Diseases Pharmacists: An Executive Summary. *Pharmacotherapy: The Journal of Human Pharmacology and Drug Therapy*.

[50] Ullah Nayan M., Sillman B., Hasan M. (2023). Advances in Long-Acting Slow Effective Release Antiretroviral Therapies for Treatment and Prevention of HIV Infection. *Advanced Drug Delivery Reviews*.

[51] Thoueille P., Choong E., Cavassini M., Buclin T., Decosterd L. A. (2022). Long-Acting Antiretrovirals: A New Era for the Management and Prevention of HIV Infection. *Journal of Antimicrobial Chemotherapy*.

[52] Spreen W. R., Margolis D. A., Pottage J. C. (2013). Long-Acting Injectable Antiretrovirals for HIV Treatment and Prevention. *Current Opinion in HIV and AIDS*.

[53] Krovi S. A., Johnson L. M., Luecke E., Achilles S. L., van der Straten A. (2021). Advances in Long-Acting Injectables, Implants, and Vaginal Rings for Contraception and HIV Prevention. *Advanced Drug Delivery Reviews*.

[54] Margolis D. A., Gonzalez-Garcia J., Stellbrink H. J. (2017). Long-Acting Intramuscular Cabotegravir and Rilpivirine in Adults With HIV-1 Infection (LATTE-2): 96-Week Results of a Randomised, Open-Label, Phase 2b, Non-Inferiority Trial. *The Lancet*.

[55] Orkin C., Arasteh K., Górgolas Hernández-Mora M. (2020). Long-Acting Cabotegravir and Rilpivirine After Oral Induction for HIV-1 Infection. *New England Journal of Medicine*.

[56] Smith G. H. R., Henry W. K., Podzamczer D. (2021). Efficacy, Safety, and Durability of Long-Acting Cabotegravir and Rilpivirine in Adults With Human Immunodeficiency Virus Type 1 Infection: 5-Year Results From the LATTE-2 Study. *Open Forum Infectious Diseases*.

[57] Mills A., Richmond G. J., Newman C. (2022). Long-Acting Cabotegravir and Rilpivirine for HIV-1 Suppression: Switch to 2-Monthly Dosing After 5 Years of Daily Oral Therapy. *AIDS*.

[58] Gaur A. H., Capparelli E. V., Calabrese K. (2024). Safety and Pharmacokinetics of Oral and Long-Acting Injectable Cabotegravir or Long-Acting Injectable Rilpivirine in Virologically Suppressed Adolescents With HIV (IMPAACT 2017/MOCHA): A Phase 1/2, Multicentre, Open-Label, Non-Comparative, Dose-Finding Study. *The Lancet HIV*.

[59] Segal-Maurer S., DeJesus E., Stellbrink H. J. (2022). Capsid Inhibition With Lenacapavir in Multidrug-Resistant HIV-1 Infection. *New England Journal of Medicine*.

[60] Gupta S. K., Berhe M., Crofoot G. (2023). Lenacapavir Administered Every 26 Weeks or Daily in Combination With Oral Daily Antiretroviral Therapy for Initial Treatment of HIV: A Randomised, Open-Label, Active-Controlled, Phase 2 Trial. *The Lancet HIV*.

[61] Perrier M., Bertine M., Le Hingrat Q. (2017). Prevalence of GAG Mutations Associated With In Vitro Resistance to Capsid Inhibitor GS-CA1 in HIV-1 Antiretroviral-Naive Patients. *Journal of Antimicrobial Chemotherapy*.

[62] Bester S. M., Wei G., Zhao H. (2020). Structural and Mechanistic Bases for a Potent HIV-1 Capsid Inhibitor. *Science*.

[63] Begley R., Lutz J., Rhee M. (2020). Lenacapavir Sustained Delivery Formulation Supports 6-Month Dosing Interval. https://www.hivandmore.de/kongresse/iac2020/Begley_LEN-Sustained-delivery-formulation-supports-q6mo-dosing_AIDS2020_PEB0265_Submitted.pdf.

[64] Chandiwana N. C., Serenata C. M., Owen A. (2021). Impact of Long-Acting Therapies on the Global HIV Epidemic. *AIDS*.

[65] Baeten J. M., Palanee-Phillips T., Brown E. R. (2016). Use of a Vaginal Ring Containing Dapivirine for HIV-1 Prevention in Women. *New England Journal of Medicine*.

[66] Gashema P., Iradukunda P. G., Saramba E. (2025). Bridging the Gap: Identifying Barriers and Strategies for Widespread Implementation of Long-Acting Injectable Antiretroviral Therapy in Sub-Saharan Africa: A Scoping Review. *BMC Infectious Diseases*.

[67] Teichner P., Chamay N., Elliot E. (2024). Cabotegravir + Rilpivirine Long-Acting: Overview of Injection Guidance, Injection Site Reactions, and Best Practices for Intramuscular Injection Administration. *Open Forum Infectious Diseases*.

[68] Kityo C., Cortes C. P., Phanuphak N., Grinsztejn B., Venter F. (2022). Barriers to Uptake of Long-Acting Antiretroviral Products for Treatment and Prevention of HIV in Low- and Middle-Income Countries (LMICs). *Clinical Infectious Diseases*.

[69] Liu Y., Cao W., Sun M., Li T. (2020). Broadly Neutralizing Antibodies for HIV-1: Efficacies, Challenges and Opportunities. *Emerging Microbes & Infections*.

[70] Fan M., Berkhout B., Herrera-Carrillo E. (2022). A Combinatorial CRISPR-Cas12a Attack on HIV DNA. *Molecular Therapy-Methods & Clinical Development*.

[71] Sok D., Burton D. R. (2018). Recent Progress in Broadly Neutralizing Antibodies to HIV. *Nature Immunology*.

[72] Thavarajah J. J., Hønge B. L., Wejse C. M. (2024). The Use of Broadly Neutralizing Antibodies (bNAbs) in HIV-1 Treatment and Prevention. *Viruses*.

[73] Gautam R., Nishimura Y., Pegu A. (2016). A Single Injection of Anti-HIV-1 Antibodies Protects Against Repeated SHIV Challenges. *Nature*.

[74] Moldt B., Rakasz E. G., Schultz N. (2012). Highly Potent HIV-Specific Antibody Neutralization In Vitro Translates Into Effective Protection Against Mucosal SHIV Challenge In Vivo. *Proceedings of the National Academy of Sciences*.

[75] Barouch D. H., Whitney J. B., Moldt B. (2013). Therapeutic Efficacy of Potent Neutralizing HIV-1-Specific Monoclonal Antibodies in SHIV-Infected Rhesus Monkeys. *Nature*.

[76] Mahomed S. (2024). Broadly Neutralizing Antibodies for HIV Prevention: A Comprehensive Review and Future Perspectives. *Clinical Microbiology Reviews*.

[77] Mahomed S., Pillay K., Hassan-Moosa R. (2025). Clinical Trials of Broadly Neutralizing Monoclonal Antibodies in People Living With HIV: A Review. *AIDS Research and Therapy*.

[78] Huyghe J., Magdalena S., Vandekerckhove L. (2017). Fight Fire With Fire: Gene Therapy Strategies to Cure HIV. *Expert Review of Anti-Infective Therapy*.

[79] Li H., Yang Y., Hong W., Huang M., Wu M., Zhao X. (2020). Applications of Genome Editing Technology in the Targeted Therapy of Human Diseases: Mechanisms, Advances and Prospects. *Signal Transduction and Targeted Therapy*.

[80] Ahmed M. M., Kayode H. H., Okesanya O. J. (2024). CRISPR-Cas Systems in the Fight Against Antimicrobial Resistance: Current Status, Potentials, and Future Directions. *Infection and Drug Resistance*.

[81] Barrangou R., Fremaux C., Deveau H. (2007). CRISPR Provides Acquired Resistance Against Viruses in Prokaryotes. *Science*.

[82] Brouns S. J., Jore M. M., Lundgren M. (2008). Small CRISPR RNAs Guide Antiviral Defense in Prokaryotes. *Science*.

[83] Schmidt J. K., Strelchenko N., Park M. A. (2020). Genome Editing of CCR5 by CRISPR-Cas9 in Mauritian Cynomolgus Macaque Embryos. *Scientific Reports*.

[84] Dash P. K., Kaminski R., Bella R. (2019). Sequential LASER ART and CRISPR Treatments Eliminate HIV-1 in a Subset of Infected Humanized Mice. *Nature Communications*.

[85] Schumann K., Lin S., Boyer E. (2015). Generation of Knock-In Primary Human T Cells Using Cas9 Ribonucleoproteins. *Proceedings of the National Academy of Sciences*.

[86] Yin L., Zhao F., Sun H. (2020). CRISPR-Cas13a Inhibits HIV-1 Infection. *Molecular Therapy-Nucleic Acids*.

[87] Liu W., Li L., Jiang J., Wu M., Lin P. (2021). Applications and Challenges of CRISPR-Cas Gene-Editing to Disease Treatment in Clinics. *Precision Clinical Medicine*.

[88] Okesanya O. J., Ahmed M. M., Ogaya J. B. (2025). Reinvigorating AMR Resilience: Leveraging CRISPR-Cas Technology Potentials to Combat the 2024 WHO Bacterial Priority Pathogens for Enhanced Global Health Security—A Systematic Review. *Tropical Medicine and Health*.

[89] Mahajan S., Choudhary S., Kumar P., Tomar S. (2021). Antiviral Strategies Targeting Host Factors and Mechanisms Obliging + ssRNA Viral Pathogens. *Bioorganic & Medicinal Chemistry*.

[90] McBrien J. B., Mavigner M., Franchitti L. (2020). Robust and Persistent Reactivation of SIV and HIV by N-803 and Depletion of CD8+ Cells. *Nature*.

[91] Fidler S., Stöhr W., Pace M. (2020). Antiretroviral Therapy Alone Versus Antiretroviral Therapy With a Kick and Kill Approach, on Measures of the HIV Reservoir in Participants With Recent HIV Infection (The RIVER Trial): A Phase 2, Randomised Trial. *Lancet*.

[92] Lewin S. R., Rasmussen T. A. (2020). Kick and Kill for HIV Latency. *The Lancet*.

[93] Vansant G., Bruggemans A., Janssens J., Debyser Z. (2020). Block-and-Lock Strategies to Cure HIV Infection. *Viruses*.

[94] Andre M., Nair M., Raymond A. D. (2023). HIV Latency and Nanomedicine Strategies for Anti-HIV Treatment and Eradication. *Biomedicines*.

[95] Méndez C., Ledger S., Petoumenos K., Ahlenstiel C., Kelleher A. D. (2018). RNA-Induced Epigenetic Silencing Inhibits HIV-1 Reactivation From Latency. *Retrovirology*.

[96] Gray G. E., Corey L. (2021). The Path to Find an HIV Vaccine. *Journal of the International AIDS Society*.

[97] Cao S., Woodrow K. A. (2019). Nanotechnology Approaches to Eradicating HIV Reservoirs. *European Journal of Pharmaceutics and Biopharmaceutics*.

[98] Mamo T., Moseman E. A., Kolishetti N. (2010). Emerging Nanotechnology Approaches for HIV/AIDS Treatment and Prevention. *Nanomedicine*.

[99] Okware S. (2024). Introductory Chapter: New Emerging Treatment Options for HIV-AIDS. *HIV Treatment-New Developments*.

[100] Zubair A., Bibi B., Habib F., Sujan A., Ali M. (2024). Clinical Trials and Recent Progress in HIV Vaccine Development. *Functional and Integrative Genomics*.

[101] Dale C. J., Kent S. J. (2000). Vaccines for HIV. *Expert Opinion on Therapeutic Patents*.

[102] Ahmad S., Baqar T., Kumar R. (2023). A Comprehensive Review on Types of Vaccines: From Classic to Cutting-Edge. *Vaccines & Vaccination Open Access*.

[103] Hokello J., Sharma A. L., Tyagi M. (2021). An Update on the HIV DNA Vaccine Strategy. *Vaccines*.

[104] Yue J., Liu Y., Zhao M., Bi X., Li G., Liang W. (2023). The R&D Landscape for Infectious Disease Vaccines. *Nature Reviews Drug Discovery*.

[105] Mann J. K., Ndung’u T. (2015). HIV-1 Vaccine Immunogen Design Strategies. *Virology Journal*.

[106] Kaur A., Vaccari M. (2024). Exploring HIV Vaccine Progress in the Pre-Clinical and Clinical Setting: From History to Future Prospects. *Viruses*.

[107] Gilbertson A., Tucker J. D., Dubé K., Dijkstra M., Rennie S. (2021). Ethical Considerations for HIV Remission Clinical Research Involving Participants Diagnosed During Acute HIV Infection. *BMC Medical Ethics*.

[108] Lee J. H., Crotty S. (2021). HIV Vaccinology: 2021 Update. *Seminars in Immunology*.

[109] Kim J., Vasan S., Kim J. H., Ake J. A. (2021). Current Approaches to HIV Vaccine Development: A Narrative Review. *Journal of the International AIDS Society*.

[110] Gray G. E., Mngadi K., Lavreys L. (2024). Mosaic HIV-1 Vaccine Regimen in Southern African Women (Imbokodo/HVTN 705/HPX2008): A Randomised, Double-Blind, Placebo-Controlled, Phase 2b Trial. *The Lancet Infectious Diseases*.

[111] Kaushik R., Kant R., Christodoulides M. (2023). Artificial Intelligence in Accelerating Vaccine Development-Current and Future Perspectives. *Frontiers in Bacteriology*.

[112] Adepoju V. A., Udah D. C., Adnani Q. E. S. (2025). Harnessing Artificial Intelligence to Revolutionize HIV Self-Testing and Broader Infectious Disease Diagnostics. *Journal of HIV*.

[113] Olawade D. B., Teke J., Fapohunda O. (2024). Leveraging Artificial Intelligence in Vaccine Development: A Narrative Review. *Journal of Microbiological Methods*.

[114] Kumar S., Jada S. K., Subhadra S., Sahu P. S. (2024). Progresses and Challenges in HIV Vaccine. *Interdisciplinary Biotechnological Advances*.

[115] Scott G. Y., Worku D. (2024). HIV Vaccination: Navigating the Path to a Transformative Breakthrough—A Review of Current Evidence. *Health Science Reports*.

[116] Smit J., Middelkoop K., Myer L. (2005). Socio-Behaviour Challenges to Phase III HIV Vaccine Trials in Sub-Saharan Africa. *African Health Sciences*.

[117] Johnson M. M., Jones C. E., Clark D. N. (2022). The Effect of Treatment-Associated Mutations on HIV Replication and Transmission Cycles. *Viruses*.

[118] Kochhar S. (2013). Challenges and Impact of Conducting Vaccine Trials in Asia and Africa. *Human Vaccines & Immunotherapeutics*.

[119] Xu W., Li H., Wang Q. (2017). Advancements in Developing Strategies for Sterilizing and Functional HIV Cures. *BioMed Research International*.

[120] Johnston R. (2016). Gene Therapy to Cure HIV: Where to From Here?. *AIDS Patient Care and STDs*.

[121] Thèze J., Chakrabarti L. A., Vingert B., Porichis F., Kaufmann D. E. (2011). HIV Controllers: A Multifactorial Phenotype of Spontaneous Viral Suppression. *Clinical Immunology*.

[122] Rodríguez-Muñoz J., Moreno S. (2019). Strategies for the Cure of HIV Infection. *Enfermedades Infecciosas y Microbiología Clínica*.

[123] Detels R., Wu J., Wu Z. (2019). Control of HIV/AIDS Can Be Achieved With Multi-Strategies. *Global Health Journal*.

[124] Okesanya O. J., Manirambona E., Buban J. M. A., Lucero-Prisno D. E. (2023). Sustaining Long-Term Pre-Exposure Prophylaxis Use Among International Migrants. *Population Medicine*.

[125] Jose S., Marfatia Y., Baxi R., Shah R. (2017). Pre- and Post-Sexual Exposure Prophylaxis of HIV: An Update. *Indian Journal of Sexually Transmitted Diseases and AIDS*.

[126] Global Health Policy (2024). The Global HIV/AIDS Epidemic. https://www.kff.org/global-health-policy/fact-sheet/the-global-hiv-aids-epidemic/.

[127] Kumah E., Boakye D. S., Boateng R., Agyei E. (2023). Advancing the Global Fight Against HIV/AIDS: Strategies, Barriers, and the Road to Eradication. *Annals of Global Health*.

[128] Lyden T. W., Vogt E., Ng A. K., Johnson P. M., Rote N. S. (1992). Monoclonal Antiphospholipid Antibody Reactivity Against Human Placental Trophoblast. *Journal of Reproductive Immunology*.

[129] Thapa S., Hannes K., Buve A., Bhattarai S., Mathei C. (2018). Theorizing the Complexity of HIV Disclosure in Vulnerable Populations: A Grounded Theory Study. *BMC Public Health*.

[130] Moffitt A. H. (2021). December 2021. *American Journal of Orthodontics and Dentofacial Orthopedics*.

[131] Janighorban M., Boroumandfar Z., Pourkazemi R., Mostafavi F. (2022). Barriers to Vulnerable Adolescent Girls’ Access to Sexual and Reproductive Health. *BMC Public Health*.

[132] Australian Government (2024). Global Health Initiatives. https://www.dfat.gov.au/development/topics/development-issues/education-health/health/global-health-initiatives.

[133] Kapadia F., Landers S. (2020). Ending the HIV Epidemic: Getting to Zero and Staying at Zero. *American Journal of Public Health*.

[134] Heath K., Levi J., Hill A. (2021). The Joint United Nations Programme on HIV/AIDS 95-95-95 Targets: Worldwide Clinical and Cost Benefits of Generic Manufacture. *AIDS*.

[135] Goldstein D., Salvatore M., Ferris R., Phelps B. R., Minior T. (2023). Integrating Global HIV Services With Primary Health Care: A Key Step in Sustainable HIV Epidemic Control. *Lancet Global Health*.

[136] Bulstra C. A., Hontelez J. A. C., Otto M. (2021). Integrating HIV Services and Other Health Services: A Systematic Review and Meta-Analysis. *PLoS Medicine*.

[137] Patel P., Rose C. E., Collins P. Y. (2018). Noncommunicable Diseases Among HIV-Infected Persons in Low-Income and Middle-Income Countries. *AIDS*.

[138] McCombe G., Lim J., Hout M. C. V. (2022). Integrating Care for Diabetes and Hypertension With HIV Care in Sub-Saharan Africa: A Scoping Review. *International Journal of Integrated Care*.

[139] Nkhoma L., Sitali D. C., Zulu J. M. (2022). Integration of Family Planning Into HIV Services: A Systematic Review. *Annals of Medicine*.

[140] Sin N. L., DiMatteo M. R. (2014). Depression Treatment Enhances Adherence to Antiretroviral Therapy: A Meta-Analysis. *Annals of Behavioral Medicine*.

[141] Safren S. A., O’Cleirigh C., Andersen L. S. (2021). Treating Depression and Improving Adherence in HIV Care With Task‐Shared Cognitive Behavioural Therapy in Khayelitsha, South Africa: A Randomized Controlled Trial. *Journal of the International AIDS Society*.

[142] Musayón-Oblitas Y., Cárcamo C., Gimbel S. (2019). Counseling for Improving Adherence to Antiretroviral Treatment: A Systematic Review. *AIDS Care*.

[143] Kaaya S., Eustache E., Lapidos-Salaiz I., Musisi S., Psaros C., Wissow L. (2013). Grand Challenges: Improving HIV Treatment Outcomes by Integrating Interventions for Co-Morbid Mental Illness. *PLoS Medicine*.

[144] Wroe E. B., Kalanga N., Dunbar E. L. (2020). Expanding Access to Non-Communicable Disease Care in Rural Malawi: Outcomes From a Retrospective Cohort in an Integrated NCD-HIV Model. *BMJ Open*.

[145] Myer L., Phillips T. K., Zerbe A. (2018). Integration of Postpartum Healthcare Services for HIV-Infected Women and Their Infants in South Africa: A Randomised Controlled Trial. *PLoS Medicine*.

[146] Broder S. (2010). The Development of Antiretroviral Therapy and Its Impact on the HIV-1/AIDS Pandemic. *Antiviral Research*.

[147] National Institute of Allergy and Infectious Diseases (NIAID) (2024). Clinical Trials at NIAID. https://www.niaid.nih.gov/clinical-trials.

[148] Bill Melinda Gates Foundation (2024). Generosity in Action. https://www.gatesfoundation.org/.

[149] Fogatry International Centre (2008). Advancing Science for Global Health. https://www.fic.nih.gov/.

[150] https://www.edctp.org/.

[151] Gaayeb L., Das A., James I. (2023). Voluntary Licensing of Long‐Acting HIV Prevention and Treatment Regimens: Using a Proven Collaboration‐ and Competition‐Based Mechanism to Rapidly Expand at‐Scale, Sustainable, Quality‐Assured and Affordable Supplies in LMICs. *Journal of the International AIDS Society*.

[152] Waning B., Kyle M., Diedrichsen E. (2010). Intervening in Global Markets to Improve Access to HIV/AIDS Treatment: An Analysis of International Policies and the Dynamics of Global Antiretroviral Medicines Markets. *Globalization and Health*.

[153] Gu W. G., Zhang X., Yuan J. F. (2014). Anti-HIV Drug Development Through Computational Methods. *The AAPS Journal*.

[154] Visan A. I., Negut I. (2024). Integrating Artificial Intelligence for Drug Discovery in the Context of Revolutionizing Drug Delivery. *Life*.

